# Effect of RNA Demethylase FTO Overexpression on Biomass and Bioactive Substances in Diatom *Phaeodactylum tricornutum*

**DOI:** 10.3390/biology14040414

**Published:** 2025-04-13

**Authors:** Yanan Yang, Min Yang, Yihang Zhou, Xiaoqian Chen, Bingyao Huang

**Affiliations:** 1 Guangxi Key Laboratory of Marine Drugs, Institute of Marine Drugs, Guangxi University of Chinese Medicine, Nanning 530200, China; yyn2025@126.com (Y.Y.); ym080896@163.com (M.Y.); zyh02162025@126.com (Y.Z.); 2 University Engineering Research Center of High-Efficient Utilization of Marine Traditional Chinese Medicine Resources, Nanning 530200, China

**Keywords:** FTO, *Phaeodactylum tricornutum*, epigenetic editing, m^6^A modification, carotenoid

## Abstract

*Phaeodactylum tricornutum* is rich in bioactive substances, rendering it valuable in nutrition and medicine. Epigenetic editing mediated by human RNA demethylase FTO can significantly increase the yields of rice and potato and offers significant potential for the genetic breeding of microalgae. This study aimed to enhance the production of certain metabolites in *P. tricornutum* via FTO-mediated epigenetic editing. Phenotypic analysis revealed that transgenic *P. tricornutum* exhibits significantly reduced RNA m^6^A modification levels and faster growth, producing markedly higher levels of lipids, proteins, and carotenoids than the wild type. Transcriptome analysis revealed 1009 upregulated genes and 378 downregulated genes. KEGG analysis demonstrated the upregulated expression of multiple key enzymes involved in long-chain fatty acid synthesis (e.g., ACSL, fabF, and fabG), carotenoid synthesis (e.g., crtQ, PDS, and PSY1), and amino acid synthesis (e.g., dapF, glyA, and aroK) in transgenic *P. tricornutum*, consistent with our phenotypic results. These results indicate that FTO can promote growth and increase the bioactive compound content in *P. tricornutum* by regulating the m^6^A modification of RNA, and further suggest that FTO has the potential to serve as a new tool for the epigenetic editing of microalgae.

## Simple Summary

*Phaeodactylum tricornutum* is rich in bioactive substances, including fucoxanthin and polyunsaturated fatty acids. Epigenetic editing mediated by the human RNA demethylase FTO has been shown to significantly increase yields in rice and potato. Our study aimed to improve the traits of *P. tricornutum* by applying FTO-mediated epigenetic editing. We successfully constructed transgenic algal strains. Transgenic *P. tricornutum* exhibits significantly reduced RNA m^6^A modification levels and faster growth, and also produces markedly higher levels of lipids, proteins, and carotenoids compared to the wild type. Transcriptome analysis revealed that the expression of numerous genes was upregulated. The Kyoto Encyclopedia of Genes and Genomes analysis demonstrated the upregulated expression of multiple key enzymes involved in long-chain fatty acid synthesis, carotenoid synthesis, and amino acid synthesis in transgenic *P. tricornutum*. These results indicate that exogenous FTO can promote the expression of numerous genes by decreasing overall m^6^A methylation levels in cells, ultimately enhancing the associated metabolic pathways. These findings suggest that FTO has the potential to serve as a new tool for the epigenetic editing of microalgae.

## 1. Introduction

*Phaeodactylum tricornutum* is an important planktonic alga that serves as a food source for many aquatic invertebrates, fish, crustaceans, and mollusks. It has three major morphotypes, namely, fusiform, triradiate, and oval. This alga is highly nutritious and medicinal because it is rich in fucoxanthin, polyunsaturated fatty acids (PUFAs), and other bioactive substances. Fucoxanthin has potent antioxidant properties that surpass those of beta-carotene, lutein, and other carotenoids [1]. It also scavenges intracellular oxygen free radicals, protects cellular and DNA integrity, significantly enhances cellular regenerative capacity, and delays aging. Moreover, it inhibits the proliferation of various cancerous cells [2,3,4,5], prevents obesity and diabetes [6], and improves cardiovascular function markers [7,8]. Fucoxanthin is widely applied to the food, skincare, and healthcare industries.

*P. tricornutum* is rich in PUFAs, include eicosapentaenoic acid (EPA) and docosahexaenoic acid (DHA), which are essential fatty acids for humans. They can prevent and treat cardiovascular diseases, and have anti-inflammatory, anti-aging, anti-coagulation, and immune system-regulatory effects. They have been applied clinically as auxiliary therapies for various cancer, skin, and geriatric diseases [9,10]. The lipid content accounts for 20~30% of the dry weight, and EPA can reach up to 30% of the total fatty acid content, which renders *P. tricornutum* an excellent candidate for EPA production [11,12,13,14]. Furthermore, this microalga offers advantages such as rapid growth, low levels of other PUFAs, ease of harvesting, and high-purity recovery [15]. *P. tricornutum* also exhibits the advantages of genetic engineering breeding, such as a compact genome (~27 Mb) and ease of genetic transformation [16,17,18,19]. Consequently, *P. tricornutum* is widely studied. The genome and proteome of *P. tricornutum* have been fully sequenced [19,20], and molecular tools for reverse genetics and cell biology have matured [21,22]. The CRISPR technology has also been applied to edit the genome in this species [23]. These developments have facilitated molecular investigation using *P. tricornutum* as a model organism.

The concept of RNA epigenetics posits that reversible chemical modifications of RNA, such as those found in DNA and histones, play crucial regulatory roles in gene expression [24]. This hypothesis was substantiated by the findings that fat mass and obesity-associated (FTO) demethylate RNA m^6^A [25], and that RNA methylation modifications are reversible. This significantly contributed to gene expression regulation and opened new avenues in RNA epigenetics. The established obesity gene FTO is mainly found in animals and serves as an RNA demethylase, regulating growth and development [25,26]. However, homologous proteins are not found in higher plants [27].

Epigenetics is the study of reversible and heritable changes in gene function without altering DNA sequences. Epigenetic phenomena include DNA methylation, genomic imprinting, maternal effects, gene silencing, nuclear dominance, non-coding RNAs, and histone PTM and RNA methylation [28]. Currently, RNA methylation is one of the most actively studied research areas. The RNA m^6^A modification by methyltransferases is dynamically reversible and can be removed by demethylases. It regulates gene expression through alternative splicing and mRNA nuclear export, stability, translation, and transcription [29,30]. This modification is found in fungi, animals, higher plants, and algae. It has mostly been studied in animals, where it plays important regulatory roles in biological processes such as stem cell differentiation, tissue development, brain memory, immune responses, and disease mechanisms. m^6^A is the most prevalent post-transcriptional modification in eukaryotes, accounting for ~80% of all RNA methylation events [29]. In contrast, few studies have investigated RNA m^6^A modifications in plants. Thus, the understanding of its regulatory role in plant organ development, cell division, development, and stress adaptation remains limited. Nevertheless, RNA m^6^A modification plays an important role in the regulation of plant growth and stress adaptation [31]. RNA demethylases, such as mRNA adenosine methylase (MTA) and FKBP12 (FK506-binding protein12 KD)-interacting protein 37 KD (FIP37), regulate apical meristem development in *Arabidopsis thaliana* [32,33]. Both ALKBH9b and and10b can demethylate the m^6^A modification of RNA, thus influencing flowering and nutrient uptake in *Arabidopsis* [34,35].

A method was proposed to enhance crop quality and resistance to adverse conditions through m^6^A editing [36]. This approach elicited a remarkable response that led to increased plant yield and biomass. The introduction of FTO into rice and potatoes facilitates the demethylation of RNA m^6^A modifications, resulting in substantial enhancements in crop yield, biomass, and stress tolerance [27]. Specifically, FTO overexpression in rice and potatoes resulted in a ~50% increase in yield. A comprehensive investigation of its molecular mechanisms determined that FTO-mediated demethylation of m^6^A facilitates chromatin remodeling and transcriptional activation, thereby resulting in the respective upregulation of ~11,000 and 7000 genes in leaves and roots, while simultaneously activating multiple signaling pathways. Consequently, this study introduced a novel form of epigenetic editing breeding technology that paved the way for an entirely new paradigm in plant breeding [27].

Epigenetic modifications are strongly associated with life activities in microalgae, which are lower plants. Numerous studies have investigated epigenetic modifications of DNA in microalgae. Four DNA methyltransferases (DNMTs 3, 4, 5, and 6) have recently been identified through bioinformatics analysis of *P. tricornutum*. A gene knockout of DNMT5a showed that its loss correlated with the global depletion of DNA methylation and the overexpression of young transposable elements [37]. The genome-wide methylation map of *P. tricornutum* was extensively investigated, revealing that ~6% of its genome exhibits mosaic methylation profiles that are strongly associated with gene expression regulation [38]. Several DNA methyltransferases have been identified in the cyanobacterium *Synechocystis* sp., including slr0214, M.Ssp6803I, and M.Ssp6803V. In particular, slr0214, M.Ssp6803II, M.Ssp6803III, and M.Ssp6803IV play crucial roles in *Synechocystis* sp. growth [39,40]. However, research on RNA methylation modifications is scarce. Multiple RNA methyltransferases have been identified in *Alexandrium tamutum*, *Amphidinium carterae, Cylindrotheca closterium*, and *Tetraselmis suecica*. These enzymes exhibit homology with ALKBH9b, ALKBH10b, MTB, and FIP37 in higher plants [41].

In summary, epigenetic modifications play critical roles in the activities of microalgae. However, the sparse investigations there are in this area lack depth. The application of FTO as an epigenetic editing tool in monocotyledonous (rice) and dicotyledonous (potato) plants suggests that this technology has broad applicability across plant species. Therefore, this technology holds significant potential for the genetic breeding of microalgae. The aim of this study was to utilize the demethylation capability of FTO protein to remove m^6^A modifications from RNA in microalgae, thereby activating specific metabolic pathway genes and enhancing the production of target metabolites.

## 2. Materials and Methods

### 2.1. Strains and Growth Conditions

*P. tricornutum* strain CCAP1055 was obtained from the Algae Culture Collection at the Ocean University of China. *P. tricornutum* cells were cultured in f/2 medium, which was prepared by filtering seawater through a 0.22-micron filters and subsequently sterilizing it at 121 °C. The final medium was obtained by adding the sterilized f/2 stock solution at a ratio of 1:1000. The diatom strain was inoculated into fresh culture medium at a ratio of 1:10 (*v*/*v*), and grown at 20 °C under a 16/8 h light/dark cycle in f/2 medium [42]. The intensity of the light was 70 μmol photons m^−2^s^−1^. A s’olid f/2 medium was prepared by adding 1% (*w*/*v*) agar to the liquid medium. *P. tricornutum* cells were harvested by centrifugation at 4000× *g* for 10 min during the exponential growth phase.

### 2.2. Plasmid Construction

The original human FTO cDNA sequence was obtained from GenBank Accession no. NP_001073901.1). Optimized *FTO* cDNA was obtained based on the codon preference of *P. tricornutum* through codon optimization technology and was referred to as *PtFTO*. Fragments of *PtFTO* were synthesized (Sangon Biotech Co., Ltd., Shanghai, China) and subsequently subcloned into the PUC-19 vector. An EcoRI-XbaI fragment encompassing *PtFTO* from PUC-19 was cloned into the polylinker region of the pPha-T1 plasmid, which enabled diatom selection for positive transformants using zeocin. The constructed vector was confirmed as being error-free by PCR amplification and DNA sequencing. The forward primer sequence 5′-GGTACCATGAAGCGTACCCCCACCGCCGAAG-3′ and the reverse 5′-TCTAGAGGGCTTGGCTTCGAGGAGCTGTC-3′ were used for PCR verification.

### 2.3. Generation of Transgenic P. tricornutum

*P. tricornutum* cells were grown for 5 days in f/2-Si liquid medium. Cells were harvested by centrifugation at 4000× *g* for 10 min and resuspended in a fresh f/2-Si liquid medium at a density of 5 × 10^8^ cells/mL. Cells were bombarded with Biolistic PDS-1000/He (Bio-Rad, Hercules, CA, USA) at a bombardment pressure of 1500 psi. Then, 3 mg gold microcarriers (Bio-Rad) were coated with 5 μg of plasmid in the presence of 50 μL CaCl_2_ (2.5 mol/L) and 20 μL of spermidine (0.1 mol/L), as outlined by the manufacturer’s instructions [43]. After bombardment, the cells were recovered in an f/2 plate for 24 h and were replated on selective plates containing zeocin (100 μg/mL). The plates were incubated at 20 °C under a 16/8 h light/dark cycle (70 μmol photons m^−2^s^−1^) for 2 weeks. The transformation was verified using PCR and sequencing techniques. The forward primer sequence 5′-GGTACCATGAAGCGTACCCCCACCGCCGAAG-3′ and the reverse sequence 5′-TCTAGAGGGCTTGGCTTCGAGGAGCTGTC-3′ were used for PCR verification.

### 2.4. Total RNA m^6^A Modification Level

RNA m^6^A levels in *P. tricornutum* were determined using an enzyme-linked immunosorbent assay (ELISA). Total RNA was extracted from *P. tricornutum* cells using an E.Z.N.A.^®^ Plant RNA Kit (Omega Biotek, Norcross, GA, USA). All RNA samples were treated with DNase I to eliminate genomic DNA contamination. The quality of the extracted RNA was spectrophotometrically assessed to confirm that the experimental requirements were met (OD, 260/280 > 1.9; 260/230 > 1.7). PolyAt mRNA was then purified using a Hieff NGS^®^ mRNA Isolation Master Kit (Yeasen Biotechnology, Shanghai, China). The global m^6^A levels of mRNA were detected using an EpiQuik m^6^A RNA Methylation Quantification Kit (Epigentek Group Inc., Farmingdale, NY, USA). All experiments proceeded as described by the manufacturer and sample analysis included 200 ng of poly(A) mRNA. Absorbance was measured at 450 nm using a microplate reader (Thermo Fisher Scientific, Waltham, MA, USA), and a standard curve was generated to calculate the m^6^A level, as described by the manufacturer’s protocol. All the assays were repeated at least three times.

### 2.5. Lipid Analysis

The relative contents of neutral lipids in the microalgae were determined by Neutral Red staining. Then, 8-day-old wild-type and transgenic *P. tricornutum* cells were collected for Nile Red staining. We dissolved 1 mg of Nile Red in 10 mL of acetone to prepare a stock solution with a concentration of 0.1 mg/mL. The cells were resuspended in 20% dimethyl sulfoxide (DMSO) and incubated in a 40 °C water bath for 10 min. Subsequently, 20 μL of Nile red stock solution was added into the 2 mL of cell suspension and incubated in the dark for 15 min at room temperature. The excitation and emission wavelengths for detection were set to 530 and 580 nm, respectively. The fluorescence intensities represented the relative contents of neutral lipids in the stained cells. All the assays were repeated at least three times.

Microalgal samples were harvested by centrifugation at 4000× *g* for 10 min, lyophilized at −40 °C for 24 h, and subsequently weighed to determine the dry weight. Total lipid extraction was performed using the solvent extraction method [44]. The freeze-dried samples were resuspended in a methanol/chloroform solution (2:1, *v*/*v*) and ultrasonicated for 1 h. A saturated NaCl solution was added to facilitate phase separation. The mixture was centrifuged at 10,000× *g* for 5 min, and the chloroform layer was collected. Chloroform was removed from the collected solution using a rotary evaporator. The residue was dried in a 60 °C oven for 3–4 h and subsequently weighed to determine the total lipid content. All the assays were repeated at least three times.

The lyophilized samples were thoroughly ground and then mixed with 5 mL of 0.5 mol·L^−1^ NaOH methanol. The mixture was ultrasonicated for 1 h and then sealed under N_2_ and reacted at 65 °C for 2 h. After cooling, 5 mL boron trifluoride–methanol (BF–M) solution (25% *w*/*w* in methanol) was added to the mixture, which was esterified at 65 °C for 20 min; then, fatty acids were extracted by shaking with hexane. Subsequently, hexane was added to the mixture, followed by shaking to facilitate the extraction. A saturated NaCl solution was used to promote phase separation, and the hexane phase was collected for fatty acid analysis. The fatty acid samples were analyzed by gas chromatography–mass spectrometry (GC–MS) (Agilent Technologies Inc., Santa Clara, CA, USA) following the method previously described [45]. All the assays were repeated at least three times.

### 2.6. Determination of Total Soluble Protein

The total protein was determined using a BCA protein assay kit (Solarbio Inc., Beijing, China). The 8-day-old wild-type and transgenic *P. tricornutum* cells were collected by centrifugation at 4000× *g* for 5 min. Then, the cells were resuspended in a buffer and lysed using an ultrasonic homogenizer (Ningbo Scientz Biotechnology Co., Ltd., Ningbo, China). The lysates were centrifuged at 10,000× *g* for 5 min at 2–4 °C, and the sedimentary impurities were eliminated. A 20 μL aliquot of the sample was added to 200 μL BCA working solution. The mixture was incubated at 37 °C for 30 min; then, the absorbance was measured at 562 nm. Protein concentrations were calculated based on a standard curve generated using bovine serum albumin (BSA). All the assays were repeated at least three times.

### 2.7. Determination of Total Carotenoids

The 8-day-old wild-type and transgenic *P. tricornutum* cells were collected by centrifugation. The cells were resuspended with DMSO and then incubated at 60 °C in the dark for 1 h until the algal cells became completely decolorized. The extract was centrifuged at 12,000× *g* for 5 min, and the supernatant was collected. The optical density (OD) of the extract was detected at 480, 649, and 665 nm using a microplate reader. Carotenoid and total chlorophyll concentrations were estimated using the Wellburn formula [46] as follows:

Ca = 12.19A_665_ − 3.45A_649_

Cb = 21.99A_649_ − 5.32A_665_

TCC = (1000A_480_ − 2.14Ca − 70.16Cb)/220

where Ca and Cb represent the concentrations of chlorophyll a and b, respectively; A_665,_ A_649_, and A_480_ represent OD values at wavelengths of 665, 649, and 480 nm, respectively; and TCC represents the total carotenoid concentration. All the assays were repeated at least three times.

### 2.8. Determination of Fucoxanthin

The 8-day-old wild-type and transgenic *P. tricornutum* cells were collected by centrifugation. After freeze-drying the cells for 48 h, 10 mL of 90% ethanol was added per 1 g of microalgae powder. The extract was obtained by ultrasonic-assisted extraction at 40 °C for 1 h. The extract was centrifuged at 12,000× *g* for 5 min, and the supernatant was collected. The supernatant was filtered through a 0.22 μm filter. The content of fucoxanthin in the extract was analyzed using high-performance liquid chromatography (HPLC). The entire process was conducted in the dark. All the assays were repeated at least three times.

### 2.9. Transcriptome Sampling and Sequencing

The 8-day-old wild-type and transgenic *P. tricornutum* cells were collected by centrifugation at 4000× *g* for 5 min. For the transcriptome analysis, both the wild-type and transgenic line 9# group consist of three sequencing samples each. Total RNA was extracted from microalgae using an E.Z.N.A. ^®^ Plant RNA Kit (Omega Biotek, Norcross, GA, USA). Genedenovo Biotechnology Co., Ltd. (Guangzhou, China) provided services for cDNA library construction, sequencing, and data analysis. Transcriptome analysis was performed using reference genome-based read mapping. The *P*. *tricornutum* reference genome (GCF_000150955.2) was obtained from the National Center for Biotechnology Information (NCBI) database. Bioinformatic analysis was performed using Omicsmart, a real-time interactive online platform for data analysis (http://www.omicsmart.com) (accessed on 20 November 2024). The reads were further filtered by fastp [47] (version 0.18.0). Bowtie2 [48] (version 2.2.8) was used to map reads to the ribosome RNA (rRNA) database, and the reads mapped to rRNA were subsequently removed. The paired-end clean reads were mapped to the reference genome using HISAT2 2.1.0 [49]. For differential expression analysis, the input data used for DESeq2 [50] software (version 1.40.0) are read counts, which are used to calculate *p*-values. Subsequently, multiple testing correction is applied to obtain the FDR. The FPKM values estimated by RSEM are utilized for calculating fold changes. Differentially expressed genes (DEGs) were assessed with up-regulation or down-regulation fold changes of ≥±2 and *p <* 0.05. All analyses were performed using the R package (version 3.30.3). Gene Ontology (GO) terms were assigned by using Blast2GO (version 3.3.5) [51]. GO classification was conducted with the OmicShare tools (http://www.omicshare.com/tools) (accessed on 20 November 2024), and the categorization results were represented as three independent hierarchies: molecular function, biological process, and cellular component. The Kyoto Encyclopedia of Genes and Genomes (KEGG) pathway annotation was performed by comparison against the KEGG database [52]. A corrected *p* ≤ 0.05 was set as the threshold to identify significantly enriched GO terms or KEGG pathways. The raw data were deposited in the National Genomics Data Center (NGDC) Genome Sequence Archive under the accession number CRA023747.

## 3. Results

### 3.1. Introduction of PtFTO into P. tricornutum

**Figure 1:**
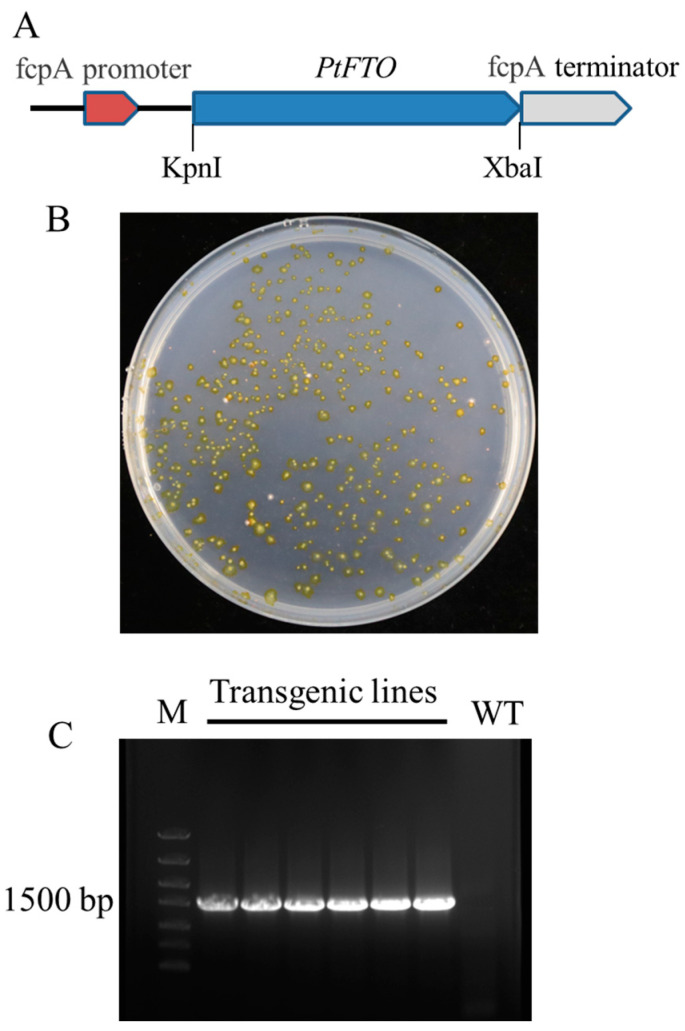
Generation of transgenic *P. tricornutum* with *PtFTO*. (**A**) Construction of transforming plasmids. *PtFTO* fragment was inserted between fcpA promoter A and fcpA terminator of pPha-T1. (**B**) Screening transformants through zeomycin resistance (100 μg/mL). Transgenic positive clones were visible after 2 weeks, and transformants were kept in F/2 medium containing 100 μg/mL zeomycin. (**C**) PCR verification of transgenic *P. tricornutum* with *PtFTO*. Specific fragments of 1518 bp were amplified from total DNA of transformants. Marker (M), transgenic lines with *PtFTO*, and wild type (WT). No *PtFTO* products were detected in WT.

The original sequence of human *FTO* cDNA was obtained from GenBank (accession no. NP_001073901.1). The optimized *FTO* cDNA was obtained using codon optimization technology according to the codon bias of *P. tricornutum* and was referred to as *PtFTO* (Table S1). The *PtFTO* spans 1518 base pairs and was resynthesized accordingly. *PtFTO* fragments were cloned into the pPha-T1 plasmid and placed under the control of the promoter *fcpA*. The constructed vector was introduced into *P. tricornutum* using a gene gun. Resistant colonies were obtained in a zeocin-selective medium (Figure 1). The transformation was verified by PCR and sequencing techniques. The PCR products were analyzed by agarose gel electrophoresis, and a 1.5-kb target fragment was successfully detected in the transgenic strains, whereas no band was present in the wild-type strain (Figure 1 and Figure S1). These results confirm that *PtFTO* was introduced into *P. tricornutum*.

### 3.2. Increased Biomass Production in Transgenic P. tricornutum

**Figure 2:**
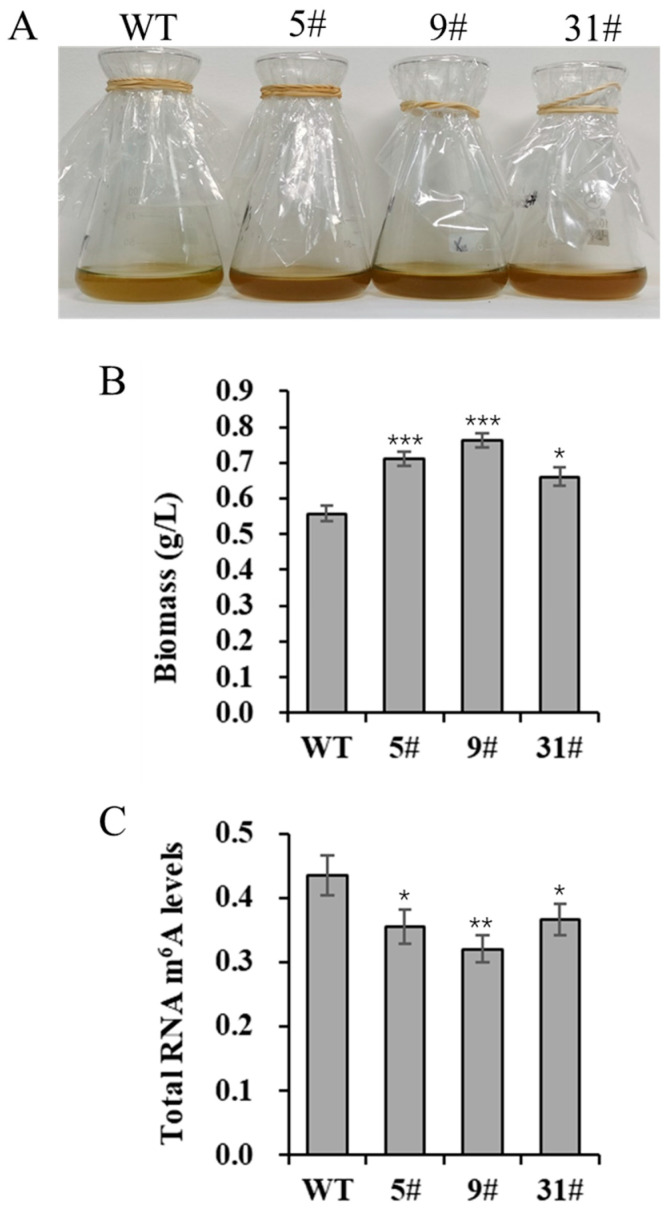
Biomass and total RNA m^6^A modification level of transgenic *P. tricornutum.* Eight-day-old *P. tricornutum* cells (WT, transgenic line 5#, 9#, and 31#) were used for all assays. (**A**) Growth phenotypic characteristics of *P. tricornutum*. (**B**) Fresh weight of *P. tricornutum*. (**C**) RNA m^6^A modification level of *P. tricornutum*. Each value represents mean ± SD (*n* = 3). Asterisks in (**B**,**C**) represent significant differences (*t* test, * *p* < 0.05; ** *p* < 0.005; *** *p* < 0.001). Results of significant analyses were compared with WT in statistical analysis.

We generated a series of primary transgenic lines via genetic transformation. Initially, independent transgenic lines were screened based on growth and neutral lipid content. Several transgenic lines (5#, 9#, and 31#) were selected for further analysis. To investigate the effects of *PtFTO* overexpression on the growth characteristics of *P. tricornutum*, we analyzed the biomass of both the transgenic and wild-type lines. The results showed that the biomass of transgenic *P. tricornutum* was higher than that of the wild type after 8 days of cultivation. Specifically, the biomass significantly increasing, rising by 27.4% and 36.9% in lines 5# and 9#, respectively, whereas the increase in the transgenic line 31# was slight (Figure 2A,B). These results suggest that *PtFTO* overexpression affects the growth and biomass accumulation of transgenic *P. tricornutum*.

### 3.3. Total m^6^A Modification of Transgenic P. tricornutum Is Significantly Decreased

m^6^A is the most abundant mRNA modification in higher eukaryotes and is reversible. In animals, the *FTO* gene encodes an mRNA demethylase that mediates RNA m^6^A demethylation. Therefore, the overexpression of *FTO* in *P. tricornutum* may affect m^6^A modification levels in the cells. In this study, the total m^6^A levels in RNA were detected by ELISA. The total m^6^A modification levels of the transgenic lines (5#, 9#, and 31#) were, respectively, 18.3%, 26.3%, and 15.7% lower than those in the wild-type cells. The extent of reduction in strain 9# was the most significant (Figure 2C). These results demonstrate that the expression of *PtFTO* can significantly decrease the total m^6^A modification level in transgenic *P. tricornutum* cells.

### 3.4. Transgenic P. tricornutum Exhibit Higher Lipid and Protein Contents

**Figure 3:**
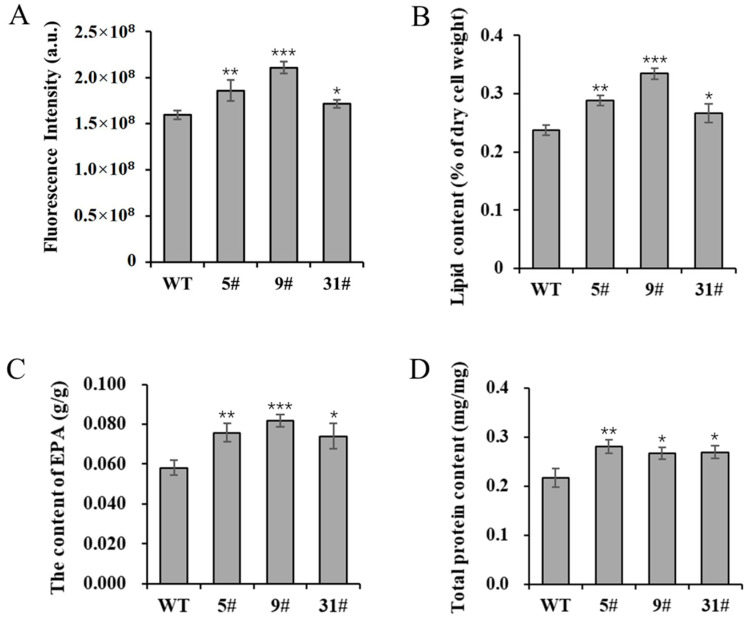
The lipid and protein contents of transgenic *P. tricornutum.* The 8-day-old *P. tricornutum* cells (WT, transgenic line 5#, 9#, and 31#) were used to all assays. (**A**) Neutral lipid content determined by Nile red staining. (**B**) Total lipid content in dry cell weight determined by gravimetry. (**C**) EPA content in dry cell weight determined by GC–MS. (**D**) Total protein content determined by BCA protein assay kit. Each value represents mean ± SD (*n* = 3). Asterisks in (**A**–**D**) represent significant differences (*t* test, * *p* < 0.05; ** *p* < 0.005; *** *p* < 0.001). The results of significant analyses were compared with WT in the statistical analysis.

The expression of *PtFTO* in *P. tricornutum* can reduce the amounts of m^6^A modification, thus potentially enhancing the expression of numerous genes in a way that subsequently affects cellular metabolic activities. In the present study, the neutral lipid content of transgenic *P. tricornutum* was determined using Nile Red staining. The results showed that the NR fluorescence intensity of transgenic lines 5# and 9# was enhanced by 16.5% and 32% compared with the wild-type variant, whereas line 31# showed a slight increase, rising by 7.4% (Figure 3A). These findings indicated a significant increase in neutral lipid levels. Total lipids were extracted from transgenic *P. tricornutum* and wild-type plants under normal culture conditions. The total lipid contents of transgenic lines 5#, 9#, and 31# were increased by 21.3%, 40.8%, and 12.4%, respectively, compared with the wild type (Figure 3B). *F. tricornutum* is an excellent candidate for EPA production. The EPA contents of transgenic lines 5#, 9#, and 31# were significantly increased, rising by 30.3%, 40.6%, and 27.2%, respectively (Figure 3C). Additionally, the total protein contents of transgenic lines 5#, 9#, and 31# were also increased by 29.7%, 23.2%, and 24.2%, respectively (Figure 3D). Collectively, these results demonstrate that overexpressed *PtFTO* promotes the accumulation of lipids and long-chain saturated fatty acids.

### 3.5. Transgenic P. tricornutum Exhibit High Carotenoid Contents

**Figure 4:**
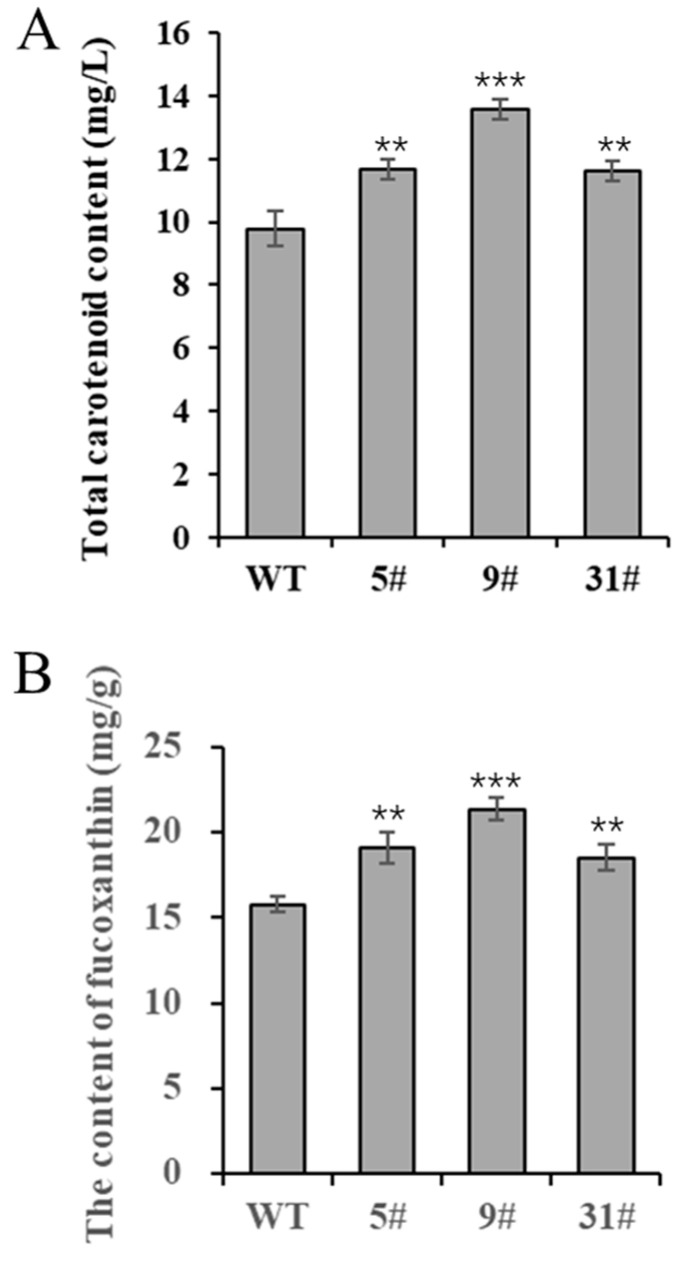
Determination of carotenoids in transgenic *P. tricornutum.* Eight-day-old *P. tricornutum* cells (WT, transgenic line 5#, 9#, and 31#) were used in all assays. (**A**) Total carotenoid content as determined by spectrophotometer. (**B**) Fucoxanthin content determined by HPLC. Each value represents mean ± SD (*n* = 3). Asterisks in (**A**,**B**) represent significant differences (*t* test, ** *p* < 0.005; *** *p* < 0.001). The results of significant analyses were compared with WT in the statistical analysis.

*P. tricornutum* is rich in carotenoids, especially fucoxanthin, and is a high-quality algal species used for the production of fucoxanthin. In this study, we measured the carotenoid content of transgenic *P. tricornutum*. Carotenoid levels were significantly higher in the three transgenic lines (particularly in line 9#) compared with the wild type, increasing by 19.2%, 38.7%, and 18.8%, respectively (Figure 4A). The trend of the fucoxanthin content in transgenic *P. tricornutum* was similar, with line 9# producing considerably significantly higher levels compared to the wild type, increasing by 21.1%, 35.4%, and 17.2%, respectively (Figure 4B). These data demonstrate that *PtFTO* expression can significantly increase the carotenoid content of *P. tricornutum*.

### 3.6. Transcriptome Analysis of Transgenic P. tricornutum

**Figure 5:**
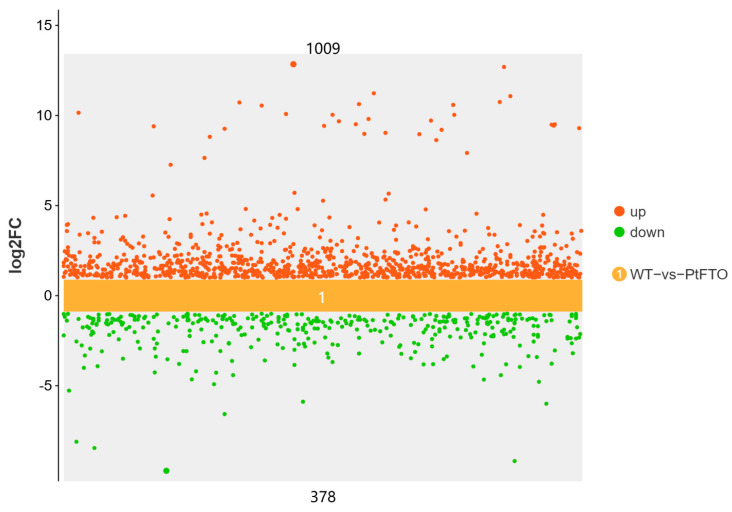
Scatter plots of DEGs between transgenic and wild-type *P. tricornutum* groups. Red dots represent up-regulated genes (1009) and green dots represent down-regulated genes (378). DEGs were enriched with up-regulation or down-regulation fold changes ≥ ±2 and *p <* 0.05.

**Figure 6:**
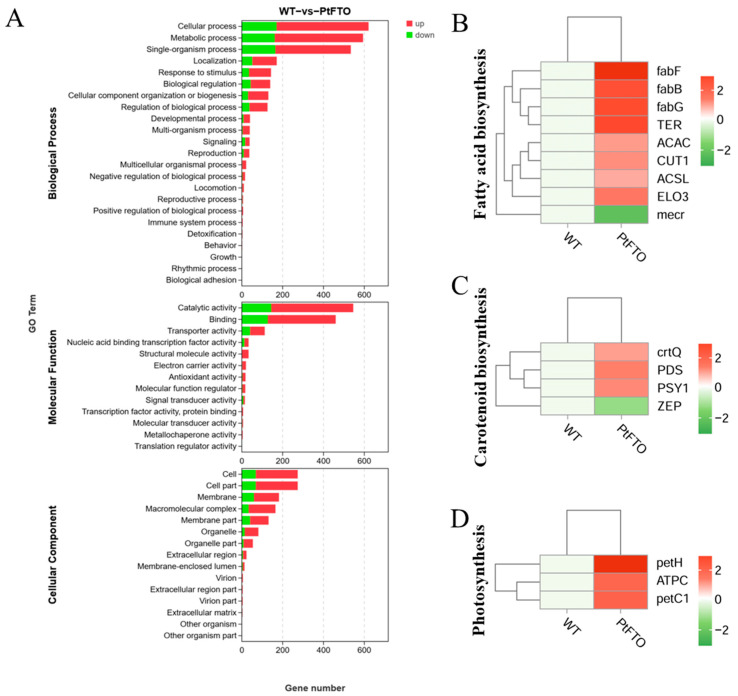
Transcriptome analysis between transgenic and wild-type *P. tricornutum* groups. (**A**) GO analysis of DEGs between transgenic and wild-type *P. tricornutum* groups. All DEGs were classified into 51 GO functional terms under the three main ontologies: biological processes, cellular components, and molecular functions. The numerical labels on the bars of column chart represent the counts of up-regulated and down-regulated genes, respectively. KEGG analysis mapped fatty acid biosynthesis (**B**), carotenoid biosynthesis (**C**), and photosynthesis (**D**). Red bars represent up-regulated genes and green bars represent down-regulated genes. DEGs were enriched with up-regulation or down-regulation fold changes of ≥±2 and *p <* 0.05.

To study the influence of the *PtFTO* gene on the expression of various genes in transgenic microalgae, transcriptome analysis technology was used to analyze the differences between transgenic *P. tricornutum* and wild type. Transgenic line 9#, which exhibited the most pronounced phenotypical change, was selected for transcriptome analysis. Based on the screening criteria of *p <* 0.05 and up-regulation or down-regulation fold changes ≥ ±1.5, we identified 2103 DEGs between transgenic and wild-type *P. tricornutum* groups, among which 1009 and 378 genes were, respectively, upregulated and downregulated (Figure 5). Based on GO analysis, all DEGs were classified into 51 GO functional terms under the three main ontologies: biological processes, cellular components, and molecular functions. In the majority of GO functional terms, significantly more genes were upregulated than downregulated (Figure 6A). These results demonstrate that the overexpression of *PtFTO* in *P. tricornutum* activated the expression of numerous genes, potentially resulting in significant changes in characteristics.

**Figure 7:**
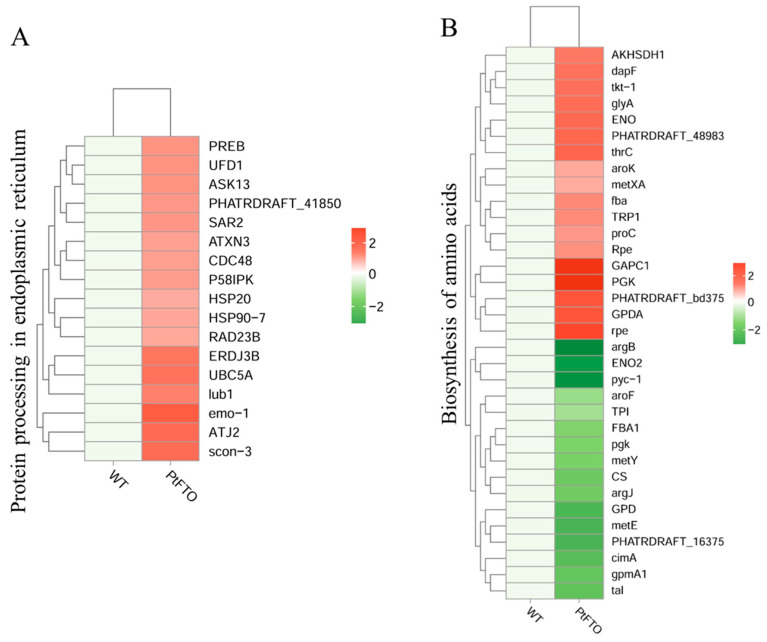
The DEGS involved in amino acid synthesis. KEGG analysis mapped protein processing in endoplasmic reticulum (**A**) and biosynthesis of amino acids (**B**). Red bars represent up-regulated genes, and green bars represent down-regulated genes. DEGs were enriched with up-regulation or down-regulation fold changes of ≥±2 and *p <* 0.05.

KEGG pathway analysis mapped multiple pathways, including the biosynthesis of amino acids, protein processing in the endoplasmic reticulum, fatty acid biosynthesis, carotenoid biosynthesis, and photosynthesis. The expression of the long-chain fatty acid synthase genes *ACSL*, *fabF*, and *fabG* in the fatty acid biosynthesis pathway of transgenic *P. tricornutum* was significantly upregulated compared with the wild-type variant. (Figure 6B). This indicated that overexpressed exogenous *PtFTO* in *P. tricornutum* modulated the expression of these genes, thus increasing the synthesis of polyunsaturated fatty acids (PUFAs) in transgenic *P. tricornutum*. Expression of the key enzymes zeta-carotene desaturase (crtQ), phytoene desaturase (PDS), and phytoene synthase (PSY1) in the carotenoid biosynthesis pathway of transgenic *P. tricornutum* was significantly upregulated compared with the wild type (Figure 6C). Additionally, the activation of photosynthetic pathways and carbohydrate metabolism might account for the accelerated growth of transgenic *P. tricornutum* (Figure 6D). Furthermore, many key genes that correlated positively with amino acid synthesis (e.g., dapF, glyA, and aroK) were significantly upregulated (Figure 7). These results are consistent with the upregulated lipid, carotenoid, and protein contents.

Transcriptome analysis revealed that exogenous RNA demethylase PtFTO plays a broad-spectrum role in *P. tricornutum*. It promotes the expression of numerous genes by decreasing overall m^6^A methylation levels in cells, ultimately enhancing the associated metabolic pathways.

## 4. Discussion

Microalgae comprise a promising biological resource with significant value for the energy, food, and medicine industries, as well as in environmental protection. Genetic breeding technology provides a critical means for the directional improvement and functional expansion of microalgae. Gene editing technology has emerged as a powerful new tool that enables the precise and efficient editing of genes in humans, other animals, and plants, thus facilitating improvements, enhancements, and the development of new characteristics [53,54,55]. Gene editing has become an important means of genetic breeding and it has been applied to various microalgae [56]. Additionally, new genetic breeding techniques are still being explored. The discovery of FTO, an RNA m^6^A demethylase, holds promise as a tool for epigenetic editing, potentially leading to the development of novel epigenetic breeding technologies aimed at enhancing crop yield. Introducing FTO into rice and potato can demethylate RNA m^6^A modifications, leading to significant increases in crop yield and biomass [27]. This discovery paves the way for novel directions in plant breeding.

Microalgae are classified as lower plant species. We speculated that FTO also functions in epigenetic editing microalgae. Therefore, we selected *P. tricornutum* as our research focus. After codon optimization, *PtFTO* was introduced into *P. tricornutum* (Figure 1). The phenotypic analysis of the transgenic *P. tricornutum* revealed a significant reduction in the abundance of RNA m^6^A modifications (Figure 2C).

RNA m^6^A is a dynamic and reversible epigenetic modification that regulates gene expression via various mechanisms such as alternative splicing, mRNA export, mRNA stability, translation, and transcriptional regulation [29,30]. The expression of FTO in rice and potatoes mediates m^6^A demethylation, thereby promoting chromatin accessibility and activating transcription [27]. In this study, transcriptome analysis demonstrated that PtFTO activated the expression of numerous genes in transgenic *P. tricornutum*, of which 1556 were upregulated (Figure 5). These findings indicate that the mechanism through which PtFTO operates in *P. tricornutum* is analogous to its function in higher plants.

Further phenotypic analysis revealed that the growth rate of transgenic *P. tricornutum* was significantly faster than that of the wild type (Figure 2A,B); moreover, the levels of bioactive compounds, such as fatty acids, carotenoids, and proteins, were also markedly increased (Figure 3 and Figure 4). These results showed that PtFTO exerts a comprehensive influence on the physiological activities of *P. tricornutum*. KEGG analysis revealed that the expression levels of key enzymes involved in long-chain fatty acid synthesis (e.g., ACSL, fabF, and fabG), carotenoid synthesis (e.g., crtQ, PDS, and PSY1), and amino acid synthesis (e.g., dapF, glyA, and aroK) were upregulated in transgenic *P. tricornutum* (Figure 6B,C and Figure 7). This upregulation might directly contribute to increased levels of fatty acids, carotenoids, and proteins and may be regulated by FTO-mediated m^6^A demethylation. Additionally, the activation of photosynthetic pathways and carbohydrate metabolism might contribute to the accelerated growth of transgenic *P. tricornutum* (Figure 6D). Therefore, similar to rice and potatoes, the expression of exogenous FTO in *P. tricornutum* may induce chromatin openness and transcriptional activation by reducing RNA m⁶A modification levels, thereby activating relevant metabolic pathways.

Based on the potential mechanisms and phenotypic changes associated with PtFTO, compared with gene editing methods, such as CRISPR/Cas, FTO-mediated epigenetic editing revealed more target sites, enabling the activation of more genes and metabolic pathways. This regulation is reversible and flexible. However, the disadvantages are significant. For example, a relatively short duration of editing effect increases the likelihood of trait degeneration. Additionally, achieving precise targeting might be challenging because it depends on RNA metabolism. Therefore, enhancing the editing efficiency and improving the genetic stability of transgenic microalgae strains are key directions for future research.

## 5. Conclusions

This study expressed *PtFTO* in *P. tricornutum* and showed that PtFTO protein reduced m^6^A methylation levels in transgenic *P. tricornutum*. Phenotypic analysis revealed significantly faster growth rates and more abundant lipids, proteins, and carotenoids in transgenic compared to wild-type *P. tricornutum*. Transcriptome analysis revealed that the expression levels of numerous genes in transgenic *P. tricornutum* are significantly upregulated. The results of our KEGG analysis revealed the upregulated expression of key enzymes involved in long-chain fatty acid (e.g., ACSL, fabF, and fabG), carotenoid (e.g., crtQ, PDS, and PSY1), and amino acid synthesis (e.g., dapF, glyA, and aroK) in transgenic *P. tricornutum*, which agreed with our phenotypic findings. These results indicated that FTO promotes growth and increases the content of bioactive compounds in *P. tricornutum* by regulating the RNA m^6^A modification. This suggests that FTO has the potential to serve as a new tool for the epigenetic editing of microalgae.

## Data Availability

The datasets generated and/or analyzed during this study are available from the corresponding author on reasonable request.

## Supplementary Materials

The following supporting information can be downloaded at: https://www.mdpi.com/article/10.3390/biology14040414/s1, Table S1. The cDNA sequence of *PtFTO*; Figure S1. The original agarose gel electrophoresis image for PCR verification of *PtFTO* transgene integration in *P. tricornutum*.
