# Supplementary material for: Effect of RNA Demethylase FTO Overexpression on Biomass and Bioactive Substances in Diatom Phaeodactylum tricornutum
Source: Biology (Basel). 2025 Apr 13;14(4):414. doi: 10.3390/biology14040414 (PMC12024952; doi:10.3390/biology14040414)
Supplement: Supplementary file 1 [file biology-14-00414-s001.zip › biology-3529651-supplementary.pdf]

Table S1. The cDNA sequence of *PtFTO.*

Gene	Optimized Sequence
PtFTO	ATGAAGCGTACCCCCACCGCCGAAGAACGTGAACGTGAAGCCAAGAAGC<br>TCCGTTTGCTCGAAGAACTCGAGGACACCTGGCTCCCCTACCTCACCCCCA<br>AGGACGACGAATTCTACCAGCAGTGGCAGTTGAAGTACCCCAAGCTCATT<br>CTCCGCGAAGCCTCCTCCGTCAGTGAAGAACTCCACAAGGAAGTCCAGGA<br>AGCCTTCCTCACCCTCCACAAGCACGGCTGTTTGTTCCGTGACTTGGTCCGT<br>ATTCAGGGAAAGGACCTCCTCACCCCCGTTTCCCGTATCCTCATTGGAAAC<br>CCCGGTTGCACCTACAAGTACCTCAACACCCGCTTGTTCACCGTCCCGTGG<br>CCCGTTAAGGGCTCCAACATTAAGCACACCGAAGCCGAAATCGCCGCCGC<br>CTGCGAAACCTTTCTCAAGCTCAACGACTACTTGCAGATTGAAACCATTCA<br>GGCCCTCGAAGAACTCGCCGCCAAGGAAAAGGCCAACGAGGACGCCGTC<br>CCCCTCTGCATGTCCGCCGACTTTCCCCGCGTCGGAATGGGTTCCTCCTACA<br>ACGGTCAGGACGAAGTGGACATTAAGTCGCGCGCCGCCTACAACGTCACC<br>CTCCTCAATTTCATGGACCCCCAGAAGATGCCTTACCTCAAGGAAGAACCC<br>TACTTCGGAATGGGAAAGATGGCTGTCTCCTGGCACCACGACGAAAACCT<br>CGTTGACCGCTCCGCCGTCGCCGTCTACTCCTACTCCTGTGAAGGACCGGA<br>AGAAGAATCCGAAGATGACTCCCACCTCGAAGGACGTGACCCGGATATTT<br>GGCACGTCGGTTTCAAGATTTCCTGGGATATCGAAACCCCCGGACTCGCCA<br>TCCCCCTCCACCAGGGAGACTGCTACTTCATGCTCGACGACTTGAACGCCA<br>CCCACCAGCACTGCGTCCTCGCCGGATCCCAGCCGCGCTTTTCCTCCACCC<br>ACCGCGTCGCCGAATGTTCCACGGGAACCCTCGACTACATTCTCCAGCGTT<br>GCCAGCTCGCCCTCCAGAACGTCTGCGACGACGTTGACAACGATGATGTTT<br>CGCTCAAGTCCTTCGAACCCGCCGTCTTGAAGCAGGGAGAAGAAATTCAC<br>AACGAAGTTGAATTTGAATGGCTCCGCCAGTTCTGGTTCCAGGGTAACCGC<br>TACCGTAAGTGCACCGACTGGTGGTGCCAGCCGATGGCCCAGTTGGAAGC<br>CCTCTGGAAGAAGATGGAAGGAGTCACCAACGCCGTCCTCCACGAAGTCA<br>AGCGTGAAGGCCTCCCCGTCGAACAGCGTAACGAAATTCTCACCGCCATTT<br>TGGCCTCCTTGACCGCCCGTCAGAACCTCCGTCGTGAATGGCACGCCCGTT<br>GCCAGTCCCGTATCGCCCGTACCCTCCCCGCCGACCAGAAGCCGGAATGC<br>CGTCCCTACTGGGAAAAGGACGACGCCTCCATGCCCTTGCCCTTCGACTTG<br>ACCGACATTGTCTCCGAACTCCGTGGACAGCTCCTCGAAGCCAAGCCCTA<br>A

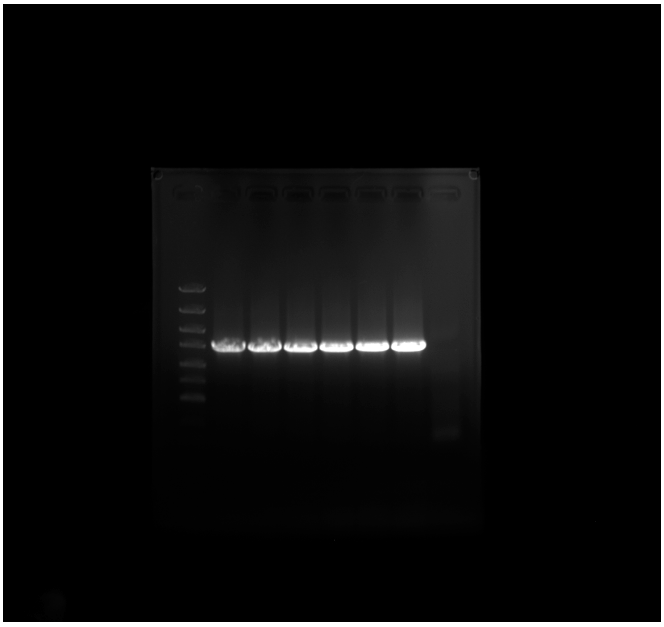

Figure S1. The original agarose gel electrophoresis image for PCR verification of *PtFTO* transgene integration in *P. tricornutum*.
